# Milk’s Flows: Making and Transmitting Kinship, Health, and Personhood

**DOI:** 10.1136/medhum-2019-011829

**Published:** 2021-05-24

**Authors:** Roslyn Malcolm

**Affiliations:** Centre for Biomedicine, Self and Society, The University of Edinburgh, Edinburgh, Edinburgh, UK

**Keywords:** breastfeeding, breastfeeding, medical anthropology, built environment, midwifery

## Abstract

Milk provides a way of thinking about how the body is enacted in science, policy and popular culture. This paper follows the currents of moral and biomedical epistemologies circulating around milk, including via notions of inheritance, the practices of wet nursing, and emerging scientific knowledge about the health-related benefits of breastfeeding. By situating milk’s flows historically and culturally it shows how constructions of milk production, lactation, and infant feeding have long served as a ‘cultural signal’ of prevailing conceptions of bodies and social identities. In so doing, it explores the simultaneous power of milk as both a source of dispositional and somatic health, and an index of customary forms of unity and division. A focus on breast milk further contributes to augmenting and expanding recent debates about the biology-society nexus in science and technology studies (STS), anthropology, and sociology. Seen within biomedicine today as a carrier of somatic signals about the environment, the article reflects on how milk is bound up in the responsibilisation of women’s bodies and the internalising of potential risks to the health of their offspring. This implies an unlimited agency for women in averting health risks and in future-proofing their children to be better than well, elides the socioeconomic, and environmental forces pragmatically limiting this assumed agency, and the distinct lack of material and inter-personal support for the perinatal period in many nations.

## Introduction

The closing scene of John Steinbeck’s *The Grapes of Wrath* offers the powerful and transgressive image of a young woman breastfeeding an aged, starving man. Having lost her own child, she acts to save the man’s life as their respective families’ shelter from a storm in an old barn. The scene is situated in the context of dustbowl USA and the broken promises of the land of milk and honey. This subversive scene is a reproduction of the Roman Charity, a variously depicted ancient Roman moral tale of a daughter who keeps her father alive by breastfeeding him after he has been incarcerated and sentenced to death by starvation.

This ancient emphasis on the uniquely life-giving power of breastmilk flows into scientific, policy and personal knowledges today. It has been claimed that “[p]ossibly, no other health behaviour can affect such varied outcomes in the two individuals who are involved: the mother and the child” (Rollins et al. 2016). The ongoing public health focus on breastfeeding in the UK and other high-income countries (HICs) reflects this import (McFadden et al. 2016). Groups such as La Leche League, likewise foreground through civic action and local relationship the vital and special qualities of breastmilk (Faircloth 2013). For many individuals, organisations, and states, there is something very special about this ‘white gold’ (Falls 2017).

This paper follows the currents of these moral and biomedical epistemologies, including via notions of inheritance, the practices of wet nursing, and emerging scientific knowledge about the health-related benefits of human milk. This article builds on existing work on bodily substance (Carsten 2011, Copeman 2019) and less normative feeding practices of informal milk sharing (Falls 2017; Palmquist and Doehler 2014; Wilson 2018) and banking (Cassidy, Dykes, and Mahon 2019; Reyes-Foster, Carter, and Hinojosa 2017). Rather than detail informal milk sharing practices, or the politics of breast vs bottle I focus on more mundane breastfeeding practices. I shift to an exploration of the simultaneity of the power of milk as both a source of dispositional and somatic health, and an index of customary forms of unity and division. I seek to make a case for how historical and cultural attention to milk, and the social connections and separations its flows can produce, provide an entry point to analyses of how the body is enacted in science, policy, and popular culture. A focus on breast milk further contributes to augmenting and expanding recent debates about the biology-society nexus in science and technology studies (STS), anthropology, and sociology (Landecker 2011; Pickersgill et al. 2013; Valdez 2018), which have tended to focus on more esoteric sites for theorising such as epigenetics rather than what are, for many, mundane forms of bodily praxis.

### Fluidity and connection

In the practices of mother-infant breastfeeding, self and other are enfolded by the feeding of one on another’s body. The breast matters both materially and semiotically. Post-partum, a child is no longer within its mother, yet the latter maintains somatic influence on the former: the mother is manifest in the child who is nourished by internalising the contents of her body, who is likewise somatically and emotionally affected by this inter-corporeal interaction (sometimes positively, sometimes adversely). In psychoanalyst Melanie Klein’s view, the “first gratification which the child derives from the external world is the satisfaction experienced in being fed” (Klein 1936 cf Sutherland 1999, 4). Speaking through Klein’s work, Sutherland emphasises a development of subjective awareness in both child and mother through shared connection and mutual bodily engagement. She describes the subjectifying qualities of being a breastfeeding mother as “I leak, therefore, I am” (Sutherland 1999).

The human milk that infants imbibe, though, does not always come from their mothers. With the advent of the breast pump, formal and informal milk sharing networks connect donors and recipients in novel ways online and via banks in cities across the Global North (see Cassidy, Dykes, and Mahon 2019; Falls 2017; Palmquist and Doehler 2014; Reyes-Foster, Carter, and Hinojosa 2017; Wilson 2018). Older practices of wet nursing, for instance, have been common throughout human cultures. Accounts of kinship ties instantiated through breastfeeding in diverse global contexts offer historical accounts of connection, and simultaneous separation, formed through milk. The Islamic practice of milk kinship is a legal institution requiring that those nursed by the same woman become ‘milk siblings’ and are therefore precluded from marriage in the same way as blood relations would be. In Islam, what is prohibited by natal (*nasab*) kinship is also equally forbidden by milk kinship (*rada’a*) (Parkes 2005). These religious and legal prohibitions remain today for communities practising cross-nursing. Accordingly, the use of milk banks is prohibited by this legal theory of kinship which finds its source in the Qu’ran: “forbidden to you are… your mothers who have given suck to you, your suckling sisters” (Fortier 2007). Milk kinship practices have been highlighted in the Hindu Kush (Parkes 2001), Muslim and Christian communities of the northern Balkans (Parkes 2004), and parts of Saharan Africa (Ensel 2002 cf Parkes 2005). Sharing milk has been evidenced as an informal practice in the ancient Mediterranean (Bradley 1991), and as a canonical impediment to marriage in particular eastern Christian churches (Parkes 2005). Breastfeeding, then, does not just reflect kinship ties: it helps to make them.

In attempts to square working mothers with breastfeeding practices and with the use of pumps, others including fathers and older relatives increasingly engage in the affective practices of feeding an infant human milk, extending the dyad of child-mother into a network of human and technological actants. When not anonymised, contemporary milk sharing practices also create new networks and kinship ties between donor and recipient (Falls 2017). However, and speaking to this articles exploration of the bounding as well as connectedness enacted by human milk, these online sharing networks are predominantly used by white, middle class women (Falls 2017; Palmquist and Doehler 2014). Societal striations guiding the flows of this milk point to the exclusionary effects of these sharing practices across class and race. Milk coagulates rather than flows in these instances, acting as boundary making phenomena.

Humans are not, of course, the only beings to produce milk. Linnaean taxonomy unifies mammals; those beings that nourish their offspring via milk from the mammary glands. Interspecies milk feeding was commonplace until the emergence of safe storage and refrigeration technologies, and was widely depicted in the artwork of the occidental middle ages (Leslie and Jackson 2018). Romulus and Remus were mythologized as being raised and suckled by a she-wolf, and ‘Wild’ Peter of Avalon was depicted as covered by a thick layer of hair - the result of having been nursed by a bear. Through wide-ranging cultural narratives and imagery, the consumption of milk at once animalises humans and humanises animals, making permeable the boundaries between humans and other mammals. In the modern period, practices of inter-mammalian infant feeding became framed as subversive, and utilised in constructing distinctions between humans and animals.

Today, if divisions are formed between particular kinds and statuses of humans, then boundaries between species are perhaps doubly underscored. Still, interspecies milk feeding remains, with cow’s milk widely consumed by infants in the guise of breast milk substitutes. Cow’s milk forms a central part of the ‘healthy diet’ required of the growing child and, for many, into adulthood. Yet, it seems only by the removal of the physical practice of feeding at the body of the animal itself – mediated by the necessary pumping and storing technologies – that drinking animal milk becomes acceptable (Jackson and Leslie 2017). A vast number of humans incorporate cow’s milk, yet share no affective connection with their bovine providers.

The trickling of perceptions of the beastliness of milk feeding into considerations of human breastfeeding practices is evident in contemporary reports of women being asked to ‘cover up’ when breastfeeding within public spaces. In the UK, for instance, it only became illegal to exclude women from public space for nursing their infants in 2010 (Griffith 2013). ‘Public’ breastfeeding practices pose a challenge to expected Euro-American female identities, including workplace expectations. Clearly, for many people, there is a ‘right’ way and a ‘wrong’ way for human milk to be consumed.

### Milk’s transmissions

Milk connects as well as signal divisions and does so by transmission: over countless generations, breastmilk has been considered to hold different properties that can be transmitted from m/other to infant. For instance, medieval medicine in Europe drew heavily on Galenic tradition which held that a mother’s own milk was the best substance for her child for its action of transmitting a substance complementary to the blood given in utero. It was believed that in utero blood was whitened into milk through *dealbation* and transmitted some of the same aspects as blood, such as resemblance (Maillet 2017).

Milk has also been judged to transmit psyche as well as soma (Fildes 1986; Ozkan et al. 2012). Notions that the milk of wet nurses could transmit (defects of) character and temperament has had implications for who was chosen to be a wet nurse. In Ancient Greece, the ideal wet nurse was defined as being Greek, brunette, and composed of a calm temper (Obladen 2012). She was not to be pregnant or menstruating, both of which were believed to spoil the milk (Baumgartel, Sneeringer, and Cohen 2016). In the European middle ages, wet nurses again should be brunette, and composed of a good disposition. They were expected to eat and drink in moderation, have already birthed a son, and hold good social standing. Sexual activity and poor diet, considered signs of moral failing and poor disposition, were believed to compromise a wet nurse’s milk and invited punishments and fines (Fildes 1988). Anecdotes that “the wet nurse’s milk carried all her physical and mental qualities, her emotions” have also been widespread in Islamic cultures (Ozkan et al. 2012). In the late middle ages, animal milk was similarly understood to transmit temperament and character making specific animals such as stubborn donkeys and flighty horses unsuitable as wet nurses (Maillet 2017). If animal wet nurses had to be used because of a lack of other preferred sources of milk, goats tended to be the preferred option.

In recent decades, conceptualisations around milk’s transmissions contain ripples of the older epistemologies noted above. Within HICs the engagement by breastfeeding women in ‘healthy behaviours’, such as eating a ‘balanced diet’ and taking ‘plenty of exercise’, is often perceived to translate into health-giving milk and resilient babies (Kolasa, Firnhaber, and Haven 2015). Breastfeeding also forms a significant part of global health strategies in low- and middle-income countries (LMICs) where the use of breastmilk substitutes (BMS) continues to rise (Rollins et al. 2016). The salience of breastfeeding to the WHO is clear from their website, and an article titled ‘Breast is Always Best, even for HIV-Positive Mothers’ (Langa 2010) noted a 2009 change in the WHO’s position to the effect that HIV-positive mothers should take antiretroviral drugs, and breastfeed “until the infant is 12 months old” (Langa 2010). This followed the results of the WHO-led Kesho Bora study which found that giving HIV-positive mothers a combination of antiretrovirals during pregnancy, delivery, and breastfeeding reduced the risk of HIV transmission to infants by 42%. The article (and guidance) served to counter prior information and policy promoting breastmilk substitute for HIV positive mothers with the aim of pushing back against emerging formula feeding cultures in LMICs (given concerns about under-nourishment and diarrhoea, for instance).

At the same time, contradictory messages exist, leading to what Carroll (2014) refers to as the “paradoxical presence” of milk. The advice from the Centres for Disease Control and Prevention on HIV and breastfeeding for mothers in the USA is that “HIV-infected mothers should not breastfeed their infants” (CDC 2018). The uncertainties produced by the HIV epidemic and related shifting policy recommendations, family, community and health worker attitudes have had significant impacts on communities' confidence in breastfeeding in contexts such as South Africa and Zambia (Arpadi et al. 2009; Coovadia et al. 2007) and have resulted in legal action and the incarceration of HIV-positive breastfeeding women in a number of contexts. Hence, a complex nexus of normative discourses and policy imperatives regarding culpability for infant (ill-)health can be discerned.

## Emergent knowledge

Contemporary biomedical research, alongside global and public health directives, have often emphasised the perceived nutritional benefits of human milk for infants. Until recently, conflicting opinion existed over whether breastfeeding delivered any significant impact in contexts where nutrition, sanitation, and healthcare were good (Victora et al. 2016). Now, though, human milk is increasingly framed as “more than just food” (UNICEF 2019, 1). It has been argued, for instance, that exclusive breastfeeding can protect against mortality and morbidity, namely via the reduction of instances of necrotising enterocolitis, sudden infant death syndrome (SIDS) (Rollins et al. 2016), upper respiratory tract infections, and otitis media (Victora et al. 2016). In a 2016 series in *The Lancet* on breastfeeding, it was reported that if exclusive breastfeeding until 6 months and continued breastfeeding until at least the age of two were universal practices, the lives of 20 000 women and 823 000 children under the age of five could be saved globally each year (ibid.).

These statistics have been increasingly cited in public and global health literatures since 2016, particularly in relation to the 2030 Sustainable Development Goals. They have been evaluated economically: one paper, for instance, titled ‘The Cost of Not Breastfeeding’ calculates a “level of avoidable mortality and morbidity (that) translates into global health system treatment costs of US$1.1 billion annually”, with “total global economic loses […] estimated to be US$341.3 billion” – i.e., “0.70% of global gross national income” (Walters, Phan, and Mathisen 2019; see also Rollins et al. 2016). As in other areas of public health (Hoeyer, Bauer, and Pickersgill 2019), the health effects of breastfeeding, then, are becoming subject to economic logics that frame value in financial terms and present what counts as those which can be counted and costed. This is very much in line with logics that see infant feeding as a market for the production and vast consumption of formula, an industry bucking downward global trends. Such framings can also depict women’s bodies as sites of potential for infant pathogenesis and as future-proofing environments for individual and societal ills.

Health-related claims are increasingly linked to recent findings regarding the immunological benefits believed to be contained in and transmitted by the consumption of human milk throughout the perinatal period and beyond. These developments in scientific knowledge are arguably driven by the billion-dollar infant formula industry that seeks to mimic human milk as closely as possible. Evoking the rhetoric and cachet of personalised and precision medicine breastmilk has been regarded as the most “exquisite personalised medicine” (Rollins et al. 2016). It is understood to transmit elements of the mother’s microbiome and own immune responses matured throughout her life, as well as multipotential stem cells which have been found to persist in infants’ bodies for some time (Ozkan et al. 2012). Human milk is now framed as “immunologically, neurologically, endocrinologically, economically, and ecologically superior” to breastmilk substitutes (McFadden et al. 2016). Via reports of milk’s power to stimulate a ‘healthy’ microbiome in babies, and enhance appetite and blood sugar regulation, breastfeeding is also being increasingly framed as a prophylactic against the march of diabetes, overweight, and obesity (Victora et al. 2019). Specific biological and inheritable properties of human milk are being isolated and made visible through biomedical technologies.

While claims that ‘breast is best’ are often promoted in biomedical, public health, and popular literatures, notions also circulate in biomedicine and beyond that breastmilk holds the potential for transmitting forms of pathogenesis via maternal disease, stress and poor diet (Thibeau et al. 2016). Uncertain reports regarding mother-to-infant flows of an array of toxic chemical compounds by human milk are emerging (Mead 2008). A lifetime of absorbing chemicals such as bisphenol A (BPA), and pesticide residues and toxic metals such as mercury have been described as concentrating within a mother’s milk supply (Rebelo and Caldas 2016; Zimmers et al. 2014). These fears are reflected in the pasteurising and screening of milk donated to banks (Cassidy, Dykes, and Mahon 2019), the flash heating and screening practices used by informal milk sharers (Reyes-Foster, Carter, and Hinojosa 2017).

As well as these exogenous compounds, a host of endogenous compounds are considered to transmit through milk. Reminiscent of much older concerns about wet-nurses, pathogenic stress hormones such as cortisol and inflammatory cytokines produced in response to social and other stresses are regarded as flowing into human milk, shaping the future experiences of breastfed new-born (Thibeau et al. 2016). Eliding the effects of poverty and stratified reproduction a mother’s ‘lifestyle’ - including levels of alcohol and illegal drug consumption - are framed in some public health messages as having the capacity to enter the milk via the blood flow and having chemical traces transmitted to the infant (Wilson et al. 2017). These emerging epigenetic courses of knowledge reflecting human milk as “more than just food” contain tributaries from historical accounts of the transmissions believed to flow between women and the infants they nourish. The mingling of this diversity of knowledges contributes to significant uncertainty regarding the benefits and risks of breastfeeding. Ultimately, they maintain the responsibilisation of mothers, and predominantly their bodies, rather than the contexts in which they live.

## Conclusions

Attending to breastfeeding in historical and cultural perspective in juxtaposition with more recent biomedical research and public health policy has highlighted how older moral and somatic epistemologies trickle into contemporary accounts. While we should not be surprised about the flowing of old theories into new, Roberts 2002 we can ask: what is at stake in the production of new knowledges about milk? In relation to studies of environmental effects on epigenetic processes anthropologist Margaret Lock has expressed concern that “the molecular endpoints […] will capture most attention, without shedding light on the complex factors implicated in the perturbations of these molecular barometers” (Lock 2013). With regards to breastfeeding, I note that an emphasis on the significance of milk, and the traces of maternal practices that it is held to contain, extends the already heavy somatic responsibility of mothers beyond birth, while the significance of the structural factors shaping the maternal body and its movement through the world can be elided.

This article has outlined perspectives on the more mundane transmission of milk between m/other and infant, rather than centring on the less common practices of informal milk sharing (see Cassidy, Dykes, and Mahon 2019; Falls 2017; Palmquist and Doehler 2014; Reyes-Foster, Carter, and Hinojosa 2017; Wilson 2018). Indeed it is the act of transmission and what connections and separations the flows of this biological substance serves to enact that are in focus here. Seeking to mimic the action of this substance, I have traced the flows of milk’s symbolic power through the historical record and reflected on its ability to both make fluid relations between people and to separate them. As shown, not only is breastfeeding stratified by race and class (Wilson 2018) so too are milk sharing practices.

Milk provides a way of thinking about how the body is enacted in science, policy and popular culture. By historically and culturally situating milk’s movements we are offered an edifying pool for reflection. I build on existing theoretical and empirical work that has underscored the connectedness produced through milk flows from m/other or wet-nurse to infant, but these simultaneously stream through striations limiting connections. There are taboos and screening practices (Carroll 2014; Palmquist and Doehler 2014; Reyes-Foster, Carter, and Hinojosa 2017 defining those for whom such transcorporeal sharing is precluded and this separating action of milks flows requires further examination. Seen within biomedicine today as a carrier of somatic signals about the environment (see relatedly Landecker 2011; Lappé and Landecker 2015), the ways in which the physiology of milk production, lactation, and infant feeding are constructed have long served as a ‘cultural signal’ of prevailing conceptions of bodies and social identities. They signal, for instance stratified reproduction (Wilson 2018), the responsibilisation of women’s bodies, and the internalising of potential risks to the health of their offspring (see relatedly Lappé 2016, Lappé et al. 2019, Valdez 2018). They imply ‘total motherhood’ an unlimited agency for women in averting health risks and in future-proofing their children to be better than well (Wolf 2011). They act to elide the socioeconomic, and environmental forces pragmatically limiting this assumed agency, and the distinct lack of material and inter-personal support for the perinatal period in many nations. Ultimately, they highlight a common lack of valuation of motherhood and reproductive energies as something deserving of support per se, beyond the economic benefits conveyed by averting risks to infant health and the march of non-communicable diseases.
